# Cationic Polymers for the Delivery of the Ebola DNA Vaccine Encoding Artificial T-Cell Immunogen

**DOI:** 10.3390/vaccines8040718

**Published:** 2020-12-01

**Authors:** Larisa I. Karpenko, Evgeny K. Apartsin, Sergei G. Dudko, Ekaterina V. Starostina, Olga N. Kaplina, Denis V. Antonets, Ekaterina A. Volosnikova, Boris N. Zaitsev, Anastasiya Yu. Bakulina, Aliya G. Venyaminova, Alexander A. Ilyichev, Sergei I. Bazhan

**Affiliations:** 1 State Research Center of Virology and Biotechnology “Vector”, Koltsovo, 630559 Novosibirsk Region, Russia; s.g.dudko@gmail.com (S.G.D.); starostina_ev@vector.nsc.ru (E.V.S.); forelat@ngs.ru (O.N.K.); antonec@yandex.ru (D.V.A.); Volosnikova_ea@vector.nsc.ru (E.A.V.); zaitsev@vector.nsc.ru (B.N.Z.); bakulina@gmail.com (A.Y.B.); ilyichev@vector.nsc.ru (A.A.I.); 2 Institute of Chemical Biology and Fundamental Medicine, Siberian Branch, Russian Academy of Sciences, 630090 Novosibirsk, Russia; eapartsin@gmail.com (E.K.A.); ven@niboch.nsc.ru (A.G.V.); 3 Department of Natural Sciences, Novosibirsk State University, 630090 Novosibirsk, Russia; 4 Laboratoire de Chimie de Coordination, CNRS, 31077 Toulouse, France

**Keywords:** Ebola virus disease, artificial T-cell antigens, DNA vaccine delivery, cationic polymers, immunogenicity

## Abstract

Background: According to current data, an effective Ebola virus vaccine should induce both humoral and T-cell immunity. In this work, we focused our efforts on methods for delivering artificial T-cell immunogen in the form of a DNA vaccine, using generation 4 polyamidoamine dendrimers (PAMAM G4) and a polyglucin:spermidine conjugate (PG). Methods: Optimal conditions were selected for obtaining complexes of previously developed DNA vaccines with cationic polymers. The sizes, mobility and surface charge of the complexes with PG and PAMAM 4G have been determined. The immunogenicity of the obtained vaccine constructs was investigated in BALB/c mice. Results: It was shown that packaging of DNA vaccine constructs both in the PG envelope and the PAMAM 4G envelope results in an increase in their immunogenicity as compared with the group of mice immunized with the of vector plasmid pcDNA3.1 (a negative control). The highest T-cell responses were shown in mice immunized with complexes of DNA vaccines with PG and these responses significantly exceeded those in the groups of animals immunized with both the combination of naked DNAs and the combination DNAs coated with PAMAM 4G. In the group of animals immunized with complexes of the DNA vaccines with PAMAM 4G, no statistical differences were found in the ability to induce T-cell responses, as compared with the group of mice immunized with the combination of naked DNAs. Conclusions: The PG conjugate can be considered as a promising and safe means to deliver DNA-based vaccines. The use of PAMAM requires further optimization.

## 1. Introduction

The Ebola virus (EBOV) causes acute severe illness that often results in lethal outcomes in the absence of therapy. The EBOV fever is one of the most dangerous viral diseases known to humanity. Periodic outbreaks of Ebola fever in the countries of central and western Africa, as well as the danger of spreading Ebola fever to other continents, contributed to the development of an effective vaccine and therapy for this dangerous pathogen [1,2,3]. To create a vaccine, different platforms were used: subunit vaccines, vaccines based on viral vectors, as well as vaccines based on nucleic acids, including mRNA and DNA vaccines [4,5,6,7,8,9]. Most vaccines under development are based on the use of the surface glycoprotein of the EBOV, since it is believed that the humoral immune response plays a major role in the formation of protective immunity against the pathogen.

Recently, reports were published that demonstrate the importance of the T-cell immune response. So, Sullivan et al. [10] investigated the efficacy of a vaccine based on the recombinant adenovirus serotype 5 (rAd5) encoding EBOV glycoprotein (GP) and showed that the protection of primates, after a lethal EBOV infection, depends on the virus-specific CD8+ cytotoxic T-lymphocytes (CTLs). In the work of Marzi et al., it was shown that CD8+ T-cells are required to protect primates vaccinated with recombinant vesicular stomatitis virus rVSV/ZEBOV-GP from a lethal dose of EBOV (Zaire) infection [11]. Rahim et al. [12] showed that simian adenovirus- and poxvirus MVA-vectored vaccines encoding cross-filovirus T-cell immunogens, based on conserved regions of the filovirus nucleoprotein, matrix and polymerase, demonstrated a protection of the BALB/c and C57BL/6J mice against high lethal challenges with Ebola and Marburg viruses, two distant members of the same family, by vaccine-elicited T-cells in the absence of GP antibodies.

Sakabe et al. examined CD8+ T-cell responses in 32 survivors of the 2013–2016 Ebola epidemic in West Africa. They identified CD8+ memory T-cells specific to the EBOV GP and NP proteins in the studied volunteers. Authors state that the virus-specific cellular response is one of the key factors contributing to the survival of humans after primary EBOV infection. On this basis, the authors believe that EBOV vaccines should stimulate not only the humoral but also the T-cell response and include not only GP but also NP of the EBOV [13].

One of the promising approaches to creating T-cell immunogens is to construct artificial polyepitope proteins. Such vaccines are a combination of a wide range of conservative CD4+ and CD8+ T-cell epitopes selected from different viral proteins and combined in one molecule. We used this approach to construct the DNA vaccine constructs pEV.CTL and pEV.Th, encoding artificial polyepitope T-cell immunogens, which are candidates for an EBOV vaccine [14]. We have shown that recombinant plasmids encoding artificial antigens cause the synthesis of corresponding mRNAs and proteins in transfected cells, as well as inducing specific responses both to CD4+ and CD8+ T-lymphocytes in immunized animals.

However, it should be noted that one of the problems of DNA and mRNA vaccines is their relatively low immunogenicity. To increase their immunogenicity, various delivery methods are used, including a gene gun, electroporation, liposomes and various cationic polymers [15,16]. A wide range of polymers have been developed, such as poly-L-lysine, polyethylenimine, poly (amido amine) dendrimers, poly (lactic-co-glycolic acid), chitosan and a number of others. However, each of these delivery methods has both advantages and disadvantages [17]. Therefore, the delivery of nucleic acids remains an area of active research. One of the promising approaches to targeted vaccine delivery is the use of cationic polymers that are capable of interacting with negatively charged nucleic acids, including mRNA and DNA.

In this work, cationic polymers such as a generation 4 polyamidoamine dendrimer (PAMAM G4) and a polyglucin:spermidine conjugate (PG) were used as delivery vehicles for the previously constructed DNA vaccine constructs pEV.CTL and pEV.Th. The design and subsequent study of the immunogenicity of the obtained complexes were carried out.

## 2. Materials and Methods

### 2.1. DNA Vaccine Constructions

**Figure 1:**
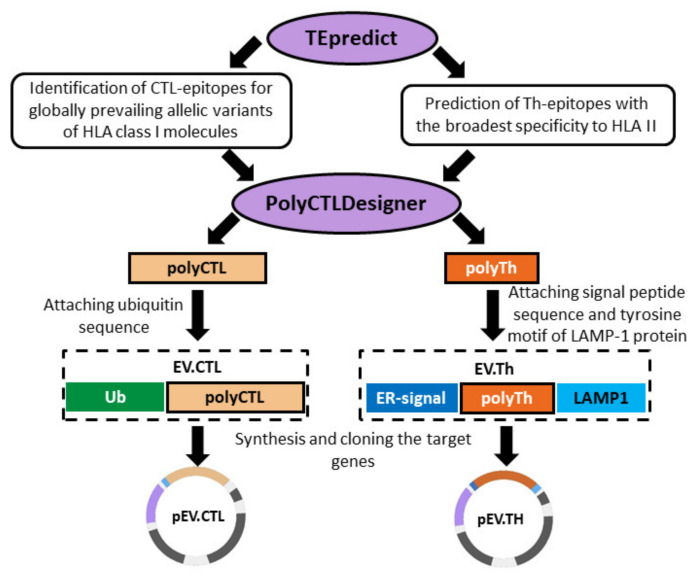
Design of artificial polyepitope Ebola virus (EBOV) immunogens EV.CTL and EV.Th.

To obtain complexes of cationic polymers with DNA, previously developed DNA vaccine constructs encoding artificial polyepitope T-cell EBOV antigens EV.CTL and EV.Th were used (Figure 1) [14]. Briefly, both immunogens were designed using conservative epitopes of the EBOV proteins GP, VP24, VP30, VP35, VP40, NP and L. To construct target immunogens, epitopes were selected from the Immune Epitope Database (http://iedb.org) [18]. We only used epitopes which have been proven to have the ability to bind to various MHC class I and II allomorphs. To design the EV.CTL antigen, CD8+ CTL epitopes were used and to design the EV.Th antigen, fragments containing CD4+ Th epitopes were used. Conservation analysis was carried out on the basis of the NCBI ProteinBank database (ncbi.nlm.nih.gov/protein), which contains the amino acid sequences of different strains of the Ebola virus. 44 CTL epitopes demonstrating a sufficiently high binding affinity for MHC class I molecules were selected. On their basis, a poly-CTL-epitope immunogen was designed using the PolyCTLDesigner software [19]. For efficient delivery to the proteasome and optimization of proteasomal cleavage, the ubiquitin sequence was added to the N-terminus of the final amino acid sequence of the poly-CTL-epitope immunogen. To design the EV.Th antigen, Th epitopes with a high binding affinity for MHC class II molecules were predicted using the TEpredict software [20]. On this basis, a poly-Th-epitope immunogen was constructed using PolyCTLDesigner. Additionally, the EBOV N-terminal GP leader peptide and the C-terminal fragment of the LAMP-1 protein were included in the construct to direct the Th antigen into the lysosome for processing and presentation through the MHC class II pathway.

The design of nucleotide sequences encoding artificial polyepitope constructs was carried out using the GeneDesigner program [21]. The developed genetic constructs (EV.CTL and EV.Th) were cloned into the vector plasmid pcDNA3.1. As a result, two recombinant DNA vaccines were designed: pEV.CTL and pEV.Th [14].

### 2.2. Cationic Polymers

**Figure 2:**
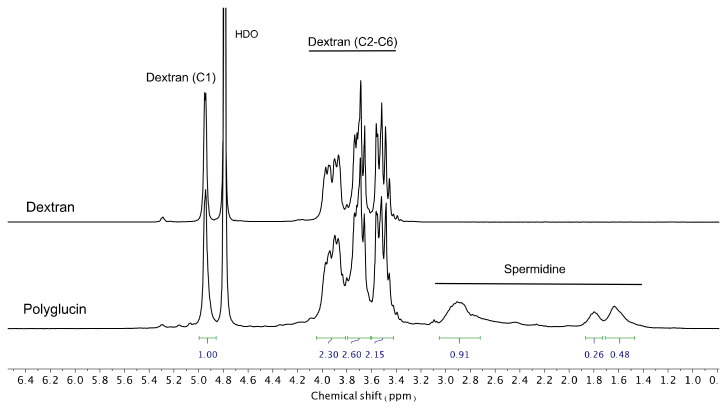
Hydrogen-1 nuclear magnetic resonance spectra (D_2_O) of Dextran 40,000 and PG.

Cationic polyglucin (PG) was obtained by conjugating polyglucin (dextran 40,000) and spermidine. Dextran 40,000 (MP Biomedicals ™, USA) was treated with sodium periodate (at the rate of 40 sodium periodate molecules per 1 dextran molecule) for 60 min in H_2_O and then gel filtration was performed on a Sephadex G-25 column in 50 mM carbonate buffer (pH 8.6). Aldehyde functions formed were quantified by a colorimetric assay (546 nm) using 2,3,5-triphenyltetrazolium salts being converted into formazan. It has been shown that one dextran molecule contains about 35–45 aldehyde groups. A spermidine solution (Sigma, USA) was added to the activated dextran at the rate of 15 spermidine molecules per one dextran molecule in 50 mM carbonate buffer (pH 8.6) and the mixture was incubated overnight. Then, sodium borohydride was added, at the rate of 80 borohydride molecules per 1 polyglucin molecule and the mixture was incubated for one hour. Unreacted components were removed by gel filtration on Sephadex G-25. Representative hydrogen-1 nuclear magnetic resonance (^1^H NMR) spectra (400 MHz, D_2_O) of Dextran 40,000 and PG recorded using Bruker AV400 spectrometer are given in Figure 2.

Diaminobutane core-based generation 4 polyamidoamine dendrimer (PAMAM G4) was purchased from Nanosynthons Inc. (USA) and used as received. To achieve good water solubility, PAMAM was dissolved with the addition of 1 M HCl up to pH~7.

### 2.3. Physicochemical Analysis of DNA-Polymer Complexes

The selection of the optimal charge ratios for DNA-polymer complexes was carried out by analyzing the degree of complexation, the sizes of the complexes and their surface charges. A series of complexes with different charge ratios were formed. DNA solutions (300 ng) were incubated with ethidium bromide solution (0.5 mM), then were mixed with polymers at room temperature (24 °C) in equal volumes on a vortex mixer V-1 plus (Biosan, Latvia). The degree of DNA binding to the polymer was determined using 1% agarose electrophoresis (45 min, 12 V/cm), by the degree of displacement of ethidium bromide by a cationic polymer (with complete displacement of ethidium bromide, the DNA band on the gel disappears). DNA fragments were visualized using a UVT-1 transilluminator (Biocom, St. Petersburg, Russia).

Size and surface charge were determined using Zetasizer Nano ZS (Malvern Panalytical, Malvern, UK). Size was measured using dynamic light scattering. The surface charge was analyzed using the method of zeta potential measurement. Measurements were run in triplicates.

### 2.4. Verification of Complex Formation before Immunization

Verification of the complexation was carried out before each immunization. For this, the negative contrast method was used [22]. The samples were examined using a JEM 1400 electron microscope (Jeol, Tokyo, Japan) at an accelerating voltage of 80 kV; photography was performed using a digital camera, Veleta (SIS, Schwentinental, Germany). Image analysis and processing were carried out using the iTEM software package (SIS, Schwentinental, Germany). At the same time, the mobility of the complexes was analyzed using a 1% agarose gel, in comparison with naked plasmids. Ethidium bromide was added to the agarose gel. 1 μg of DNA (or a complex of DNA-cationic polymer) was added to the gel groove and kept for 90 min.

### 2.5. Immunization

All experimental procedures were conducted in accordance with the recommendations in the “Guide for the Care and Use of Laboratory Animals.” The protocols were approved by the Institutional Animal Care and Use Committee (IACUC) affiliated with the State Research Center of Virology and Biotechnology “Vector” (Permit Number: SRC VB “Vector”/11-10.2019 №90).

To assess the immunogenicity of the constructed DNA complexes with cationic polymers, mice of the inbred BALB/c line (female), 16–18 g, were used. The mice were divided into 4 groups:

∑DNA group—mice immunized with a mixture of DNA plasmids pEV.CTL and pEV.Th encoding CTL and Th epitopes of the Ebola virus, respectively;
Group ∑DNA+PG—mice immunized with a mixture of complexes formed from the conjugate polyglucin:spermidine with DNA plasmids pEV.CTL and pEV.Th;
Group ∑DNA+PAMAM—mice immunized with a mixture of complexes formed from the generation 4 polyamidoamine dendrimer with DNA plasmids pEV.CTL and pEV.Th;
Group pcDNA3.1—mice immunized with the vector plasmid (a negative control).

All mice were immunized three times with an interval of two weeks, with intramuscular injection of the drug at the rate of 100 μg of each DNA per mouse. In all cases, DNA vaccine constructs pEV.CTL and pEV.Th (both naked and in complex with cationic polymers) were administered together in a volume of 200 μL.

### 2.6. ELISpot and ICS

The immunogenicity of the resulting vaccine constructs was assessed using two methods: IFN-γ ELISpot and Intracellular cytokine staining (ICS). Spleens were collected two weeks after the 3rd immunization. Splenocytes were isolated by mechanical disruption and filtration through a 40 μm cell strainer (BD Falcon™). Red blood cells were lysed by treating ACK Lysis Buffer (Sigma), splenocytes were washed in PBS and suspended in RPMI 1640 medium supplemented with 10% PBS, 2 mM L-Gln, 50 mkg/mL gentamycin. The number of cells in each spleen sample was counted using TC20™ Automated Cell Counter (Bio-Rad). About 1 × 10^8^ white blood cells were usually found in mice spleen.

ICS was carried out according to the standard protocol of BD Biosciences. Splenocytes (2 × 10^6^/well) were cultured in a round-bottom 24-well plate in 1 mL culture medium. Antigen-specific T-cells were identified by the stimulation of splenocytes with a mix of synthetic peptides corresponding to murine CTL- and Th-epitopes, included in the sequences of designed immunogens (Table 1) and by performing intracellular cytokine staining for IFN-γ and IL-2. Peptides were synthesized by Synpeptide Co., Ltd. (Shanghai, China) with >80% purity. Each peptide was added at the concentration of 10 µg/mL per 1 × 10^6^ cells, then cells were incubated for 20 h at 37 °C in 5% CO_2_ and for an additional 6 h with Golgi Plug. Cells were washed with PBS and permeabilized with Cytofix/Cytoperm™ Plus Fixation/Permeabilization Kit (BD Biosciences, USA). The following monoclonal antibodies from BD Pharmingen were used: FITC Rat Anti-Mouse CD8a, PerCP Rat Anti-Mouse CD4, PE Rat Anti-Mouse IL-2 and APC Rat Anti-Mouse IFN-γ. The samples were analyzed using the flow cytometer FACSCalibur (Becton Dickinson). A minimum of 50,000 events were acquired for each sample. Results processing was conducted using BD FACSCalibur software.

Enzyme-Linked ImmunoSpot (ELISpot) was performed using Mouse IFN-γ ELISPOT Set (BD, cat 551083, San Diego, CA, USA) according to the manufacturer’s instruction. Plates were coated with anti-mouse IFN-γ mAb and blocked with RPMI. Splenocytes were plated and stimulated with the peptide pool at a concentration of 20 μg/mL of each peptide per 1 × 10^6^ cells. Plates were incubated for 20 h at 37 °C in 5% CO_2_ and then washed with PBS. Secreted IFN-γ was detected using biotinylated anti-mouse IFN-γ mAb, streptavidin-horseradish peroxidase and AEC substrate. The numbers of IFN-γ-producing cells were calculated using an ELISpot-reader (Carl Zeiss, Göttingen, Germany).

### 2.7. Statistical Analysis

Statistical analysis of the obtained results was carried out with the R software environment for statistical analysis (version 3.3; https://www.R-project.org/). Pair-wise distribution comparison of the analyzed indices in the experimental and control groups was conducted using two-sided Mann–Whitney test. The FDR procedure was performed to correct p-values for multiple testings. The measurements were carried out in 2–4 repetitions and the median values were used for statistic processing.

## 3. Results

### 3.1. DNA Complexation with Polycationic Adjuvants

**Figure 3:**
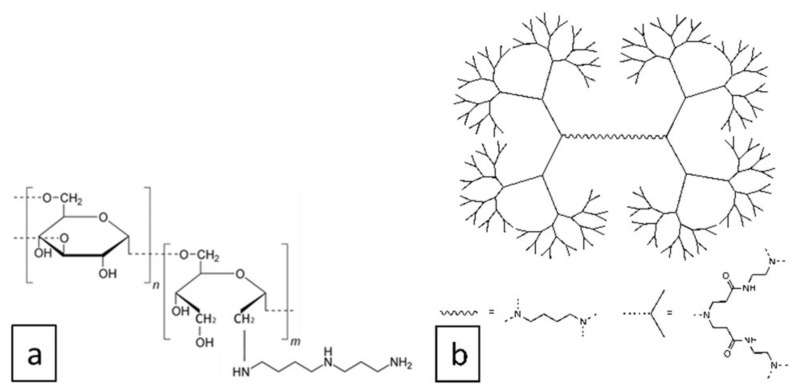
Structures of cationic polyglucin (**a**) and generation 4 polyamidoamine dendrimers (**b**) used in this work.

Previously, we designed two DNA vaccine constructs, pEV.CTL and pEV.Th, encoding artificial T-cell immunogens containing CTL and Th epitopes of EBOV proteins (Figure 1) [14]. The immunogenicity of the resulting vaccine constructs was evaluated in BALB/c mice. Their combination has been shown to induce statistically significant specific responses of both CD4+ and CD8+ T-lymphocytes in immunized animals [14]. It should be noted, however, that naked DNA has a relatively weak immunogenicity. To increase the immunogenicity of DNA vaccines, the plasmids pEV.CTL and pEV.Th were complexed with two types of polycationic polymers: cationic polyglucin and PAMAN 4G (Figure 3).

The PG–conjugate of dextran-40 with spermidine is a biocompatible and biodegradable biopolymer-derived compound. It was successfully used for the delivery of dsRNA, DNA vaccines and proteins [23,24,25,26]. Recently, we used PG for the assembly of a VLP-based vaccine against HIV—CombiHIVvac, which has passed preclinical and phase I clinical trials and has been shown to be effective in inducing B- and T-cell immune responses [27,28].

PAMAM dendrimers are commercially available, highly symmetric hyperbranched macromolecules bearing primary amino groups on the periphery, thus are able to form multiple electrostatic interactions with nucleic acids. PAMAM dendrimers are extensively used in nanomedicine [29], in particular as carriers for low-molecular drugs, DNA, RNA, proteins and so forth [30,31,32]. They do not possess their own immunogenicity [33], however, serve as efficient adjuvants for DNA vaccines [34,35]. It has been reported that PAMAM dendrimers can be toxic but they are quite biocompatible at the organism level at concentrations relevant for drug delivery [36,37].

**Figure 4:**
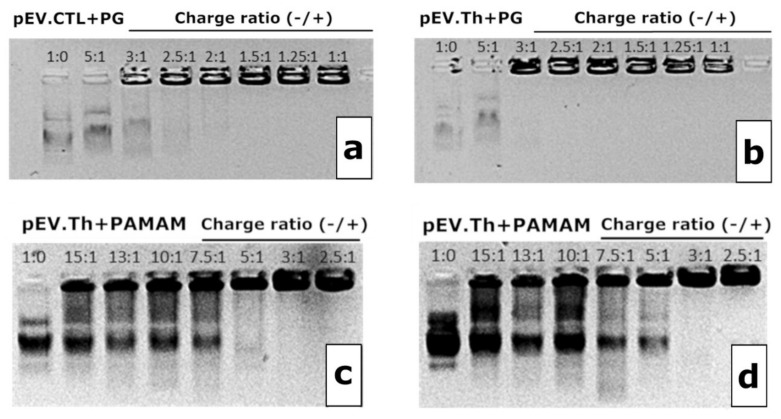
1% Agarose gel electropherograms of pEV.CTL and PG complexes (**a**), pEV.Th and PG complexes (**b**), pEV.CTL and PAMAM complexes (**c**) and pEV.Th and PAMAM complexes (**d**). N:P ratio is indicated on the graphs.

The complexation of the DNA vaccine plasmids pEV.CTL and pEV.Th with polycationic adjuvants was initially assessed by agarose gel electrophoresis (Figure 4). The binding is considered complete when the DNA is fully retained at the start line. Several charge ratios (N:P) of the negative (N) charges, that is, phosphate groups in DNA, to the positive (P) charges, that is, peripheral amino groups in cationic polymers (3:1, 2.5:1, 2:1 for PG and 5:1, 3:1 for PAMAM), have been deduced from the electrophoresis data and the properties of complexes formed were further studied.

**Figure 5:**
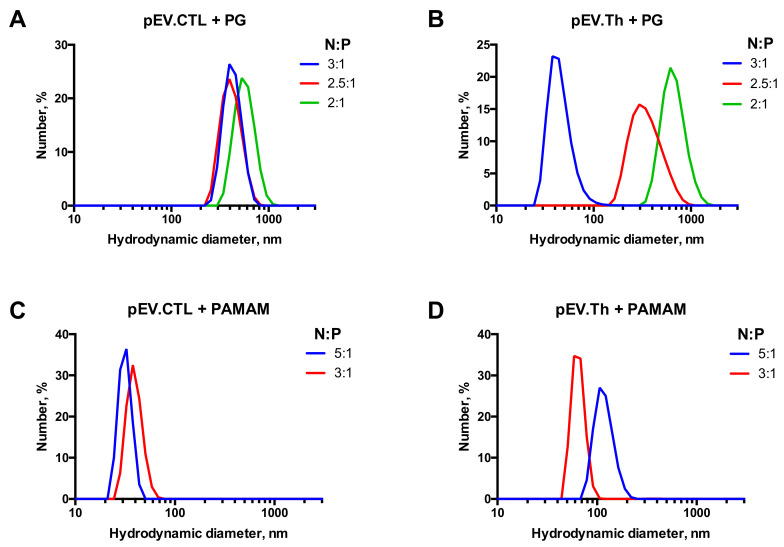
Size distribution profiles of nanoconstructions formed by DNA vaccines pEV.CTL and pEV.Th upon complexation with PG (**A**,**B**) and PAMAM G4 (**C**,**D**).

The complexes’ size evolution profiles at the N:P ratios chosen above are shown in the Figure 4. Considering both the completeness of the DNA binding and the sizes of complexes formed, the following ratios have been chosen for biological testing: 2.5:1 for PG and 3:1 for PAMAM. It is worth noting that since the DNAs were taken in the charge excess, the surface charge of the formed constructions remain negative after complexation (Table 2). The complexes formed are nanosized constructions with 100–300 nm for PG and <100 nm for PAMAM (Figure 5).

### 3.2. Immunogenicity of Cationic Polymer Complexes with DNA Vaccine Constructs

**Figure 6:**
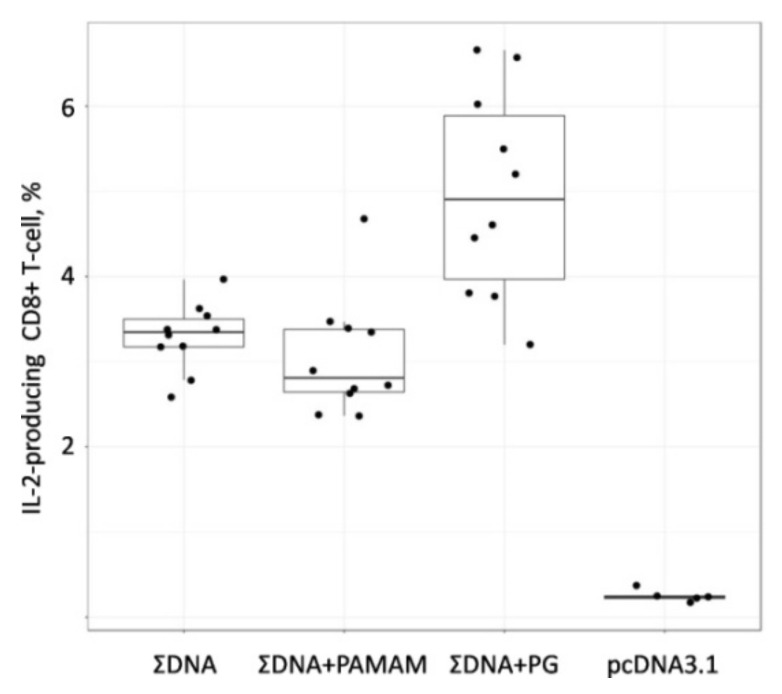
The number of T-cell obtained by IFN-γ-ELISpot assay in immunized BALB/c mice. Mice were immunized with naked plasmids or complex plasmids with cationic polymers three times with two weeks interval. After two weeks after last immunization splenocytes were collected and ELISpot analysis were performed. To analyze Ebola-specific cellular responses splenocytes were stimulated with pool of synthetic peptides, corresponding to CTL- and Th-epitopes of Ebola virus proteins. The reactivity of splenocytes was confirmed by Concanavalin A (ConA) treatment (10 mkg/mL). The number of cells per 1 × 10^6^ splenocytes represent the amount of cells expressing IFN-γ upon re-stimulation. Dots represent individual animals. ∑DNA—mice immunized with a combination of DNA plasmids pEV.CTL and pEV.Th; ∑DNA+PG—mice immunized with a combination of complexes of the conjugate polyglucin:spermidine with DNA plasmids pEV.CTL and pEV.Th; ∑DNA+PAMAM—mice immunized with a combination of complexes PAMAM 4G with DNA plasmids pEV.CTL and pEV.Th; pcDNA3.1—mice immunized with vector plasmid. Statistical analysis was performed with pairwise comparison using two-sided Mann-Whitney test implemented in R statistical software.

**Figure 7:**
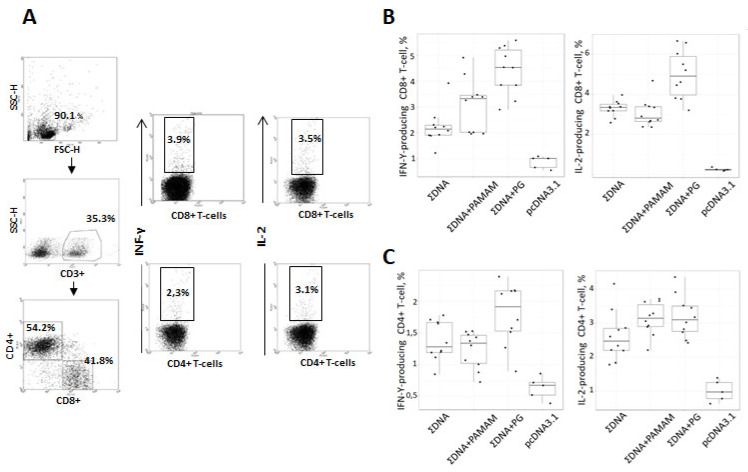
Intracellular cytokine staining with flow cytometry analysis of INF-γ and IL-2 expressing CD8+/CD4+ splenic T cells stimulated with pool of synthetic peptides, corresponding to CTL- and Th-epitopes of Ebola virus proteins. Mice were immunized with naked plasmids or complex plasmids with cationic polymers three times with two weeks interval. After two weeks after last immunization splenocytes were collected and ICS were performed. (**A**) Gating strategy for intracellular cytokine staining. The gating strategy includes selection of living cells and separation of CD8+ and CD4+ T cells within CD3+ splenocyte populations. Respectively gated T-cell populations were then analyzed for expression of INF-γ or IL-2. (**B**,**C**) Diagrams showing the number of Ebola-specific INF-γ and IL-2 expressing CD8+ T-cell (**B**) and CD4+ T-cell (**C**) responses after in vitro stimulation with a mix of specific peptides as described in Materials and methods. ∑DNA—mice immunized with a combination of DNA plasmids pEV.CTL and pEV.Th; ∑DNA+PG—mice immunized with a combination of complexes of the conjugate polyglucin:spermidine with DNA plasmids pEV.CTL and pEV.Th; ∑DNA+PAMAM—mice immunized with a combination of complexes PAMAM 4G with DNA plasmids pEV.CTL and pEV.Th; pcDNA3.1—mice immunized with vector plasmid. Statistical analysis was performed with pairwise comparison using either two-sided Mann-Whitney test implemented in R statistical software.

To study the immunogenicity of the obtained complexes, mice were immunized with DNA vaccine constructs without an envelope and coated with PG or PAMAM 4G. The dose of DNA vaccine per mouse in all constructs was the same and was 100 μg/animal. Before each immunization, the success of the complexation was checked by analyzing the mobility of particles in an agarose gel. The immunogenicity of the resulting vaccine constructs was assessed by their ability to induce T-lymphocyte responses in BALB/c mice, 14 days after the third immunization. The level of immune responses was determined using two methods, ELISpot (Figure 6) and ICS (Figure 7).

The IFN-γ-ELISpot assay data presented in Figure 6 demonstrates that in groups of animals immunized with both naked DNA and a combination of PG (ΣDNA PG) and PAMAM 4G (ΣDNA PAMAM) coated DNAs, the T-lymphocyte responses were significantly higher than in the control group of animals (pcDNA3.1) (*p* = 0.019, *p* = 0.004 and *p* = 0.006, respectively). The highest response was in the group of animals immunized with the combination of DNA vaccines with PG (ΣDNA PG). The number of IFN-γ-producing T-cells in this mice group was significantly higher than in the groups of animals immunized with naked DNA (ΣDNA) (*p* = 0.031). As for animals immunized with the combination of DNA vaccines coated with PAMAM 4G (ΣDNA PAMAM) the number of IFN-γ-producing T-cells in this mice group did not differ from those in animals immunized with the combination of naked DNAs.

To identify the T cell populations that produced IFN-γ and IL-2, we used ICS. Flow cytometric analysis of splenocytes stimulated by a pool of specific Ebola virus peptides, which are part of the designed antigens, showed that the engineered DNA vaccine induces populations of virus-specific T-lymphocytes with the phenotype (CD8+INF-γ+ and CD8+ IL-2+( and (CD4+INF-γ+ and CD4+IL-2+). Moreover, both CD8+ and CD4+ T-cells producing INF-γ and IL-2 were found in all experimental groups and showed a statistical difference in comparison with the negative control (pcDNA 3.1). The highest responses of CD8+ T-lymphocytes producing both cytokines (INF-γ and IL-2) were shown in mice immunized with complexes of DNA vaccine with PG (Figure 7) and these responses significantly exceeded those in the groups of animals immunized with both the combination of naked DNA vaccine constructs (*p* = 0.001 and 0.003, respectively) and the combination DNAs coated with PAMAM 4G (*p* = 0.021 and 0.001, respectively). In the group of animals immunized with complexes of the DNA vaccine with PAMAM 4G, no statistical differences were found in the ability to induce responses of CD8+ T-lymphocytes producing both cytokines (INF-γ and IL-2), as compared with the group of mice immunized with the of naked DNA vaccine constructs.

Regarding CD4+ T cells producing INF-γ and IL-2 their responses significantly exceeded those in the group of animals immunized vector plasmid pcDNA3.1 (*p* = 0.001) but these responses did not differ statistically between experimental groups ΣDNA, ΣDNA+PAMAM and ΣDNA+PG (Figure 7C). However, when comparing the median values, the responses CD4+ T-cells producing INF-γ and IL-2 were the highest in the ∑ДНК+PG group.

## 4. Discussion

EBOV DNA vaccines are a promising alternative to traditional vaccines based on viral vectors, recombinant proteins, peptides and attenuated or inactivated viruses. Several EBOV DNA vaccines have passed preclinical and phase I clinical trials [38,39]. The results of these studies showed that Ebola DNA vaccines are a viable platform and deserve further development.

The use of plasmid DNA for the delivery of immunogens has many attractive features, including ease of construction, low cost of production, lack of reactogenicity, induction of both CD4+ and CD8+ T cell responses and antibodies. However, the immunogenicity of DNA vaccines in the form of a naked plasmid is weak due to the DNA degradation by nucleases and the ineffectiveness of their delivery to antigen-presenting cells (APCs). There are various ways to increase the immunogenicity of DNA vaccines. Electroporation and gene gun delivery can significantly improve the overall efficacy and immunogenicity of DNA vaccines by locally delivering naked DNA to various tissues, enhancing transfection efficiency and increasing gene expression [40]. Another potentially important way to improve the efficiency of a DNA vaccine is targeting DNA to lymph nodes. Indeed, novel lipid nanoparticles have been discovered that can deliver plasmid DNA to the draining lymph node, thereby significantly increasing transfection and gene expression in this target organ [41].

An important step in the design of DNA vaccines that is not yet fully accomplished is the packaging of plasmid DNA with biocompatible and biodegradable materials. DNA complexation drives its efficient internalization into APCs and increases both humoral and cell immune responses. Herein, we used cationic polymers, namely PG and PAMAM G4 for the packaging of DNA vaccines and to increase of their efficiency. To obtain target complexes we used priory developed DNA vaccine constructions encoding artificial EBOV T-cell antigens.

The polyglucin:spermidine conjugate was chosen because we successfully used it to deliver dsRNA, DNA and proteins [23,24,25,26]. The advantages of PG include its biocompatibility and biodegradability. PG is a high-molecular dextran, which is a branched homopolysaccharide containing α-D-glucose residues. Its biodegradable properties allow its use in medical products, that is why it is used for the preparation of polyfunctional plasma-replacement solutions. The polyglucin:spermidine conjugate is a component of the HIV-1 DNA vaccine and its safety has been confirmed in the preclinical studies and Phase I clinical trials [27,28].

The PAMAM dendrimers as a DNA carrier were chosen due to the fact that it was successfully used previously for the delivery of DNA and RNA vaccines [8,42]. The packing of nucleic acids occurs due to non-covalent interactions of the sugar-phosphate backbone of the nucleic acid with amino groups in the cationic polymer. The degree of packing depends on the ratio of the size and charge of the nucleic acid to the polycation; therefore, the packing conditions must be selected in each case. DNA-containing constructions can be devoured by the cells by means of endocytosis, which is the main mechanism for its penetration into the APC.

We have shown that the complexation of DNA vaccine constructions with PG (ΣDNA+PG) leads to an increase in their immunogenic properties and causes statistically significant responses of CD8+ T-lymphocytes producing IFNγ and IL-2 in BALB/c mice, in comparison with the responses in the control group (pcDNA3.1) and in the group of mice immunized with naked DNA (ΣDNA). In the case of CD4+ T-helper lymphocytes, there was no significant difference between the naked DNA (ΣDNA), PAMAM (ΣDNA+ PAMAM) and PG (ΣDNA+PG) groups, however, when comparing the median values, the responses CD4+ T-cells producing INF-γ and IL-2 were the highest in the ∑ДНК+PG group. (Figure 7C). As for PAMAM group, a slight increase in the response of IL-2 producing CD4+ T cells (Figure 7C) could cause a slight increase in the response of IFN-γ producing CD8+ T cells in this mouse group (Figure 7B). Overall, the use of PAMAM as packaging agent did not lead to a significant improvement of the DNA vaccine immunogenicity (Figure 6 and Figure 7).

Observed differences in immunogenicity between the ΣDNA+PG and ΣDNA+PAMAM complexes can be explained by differences in the size:charge ratio of the DNA-polymer complexes. DNA complexation with both types of cationic polymers resulted in the formation of anionic particles, however, ΣDNA+PAMAM complexes were smaller than ΣDNA+PG ones and possessed a higher surface charge (Figure 4). This possibly affected the interactions with APCs.

There are several parameters that may influence the recognition and endocytosis of DNA vaccine constructions by immune cells [43]. In regard to the size and charge of constructions, there is no exact recommendation for which particle size is preferential for stimulating the uptake of DNA-dendrimer constructions by APCs. Some of the data published so far is controversial at this point [44,45,46]. Furthermore, in the case of dendrimer-mediated nucleic acid delivery into cells, its efficiency is known to vary depending on the dendrimer architecture and generation, surface charge, surface density of functional groups, molar/charge dendrimer-nucleic acid ratio, as well as cell type [42,47,48,49]. However, there is no consensus on the input of each of the parameters into the overall effect. Our findings suggest that the size and charge parameters of DNA-dendrimer complexes are likely underestimated and are important parameters defining the complexes activity. They should be indispensably taken into account while designing constructions for polycation-mediated DNA vaccine delivery. 

## 5. Conclusions

In this work, methods of T-cell immunogen delivery in the format of a DNA vaccine using PAMAM G4 dendrimers and a polyglucin:spermidine (PG) conjugate have been investigated. The optimal conditions for the formation of DNA vaccine constructs encoding the target immunogens pEV.CTL and pEV.Th, with cationic polymers, PG and PAMAM G4 were selected. Sizes, mobility and surface charge of pEV.CTL and pEV.Th complexes with PG and PAMAM were defined. It has been shown that packaging DNA vaccine constructs in the PG envelope leads to an increase in their immunogenic properties and causes a statistically significant response of virus-specific CD8+ T-lymphocytes producing IFNγ- and IL-2 in BALB/c mice. Thus, the conjugate of PG with spermidine can be considered as a promising and safe means of delivery of vaccines based on nucleic acids.

## Figures and Tables

**Table 1:** Peptides used to stimulate immunized mice splenocytes.

CTL Peptides	Th Peptides
KFINKLDALH	FKRTSFFLWVIILFQRTFSIPLGVIHNSTLQVSDVDKL
NYNGLLSSI	TNTNHFNMRTQRVKEQLSLKMLSLIRSNILKFINKLDA
PGPAKFSLL	LTLDNFLYYLTTQIHNLPHRSLRILKPTFKHASVMSRL
YFTFDLTALK	TQTYHFIRTAKGRITKLVNDYLKFFLIVQALKHNGTWQAE
LFLRATTEL	WDRQSLIMFITAFLNIALQLPCESSAVVVSGLRTLVPQSD
EYLFEVDNL	ESADSFLLMLCLHHAYQGDYKLFLESGAVKYLE
LYDRLASTV	

**Table 2:** Surface charge of DNA-carrier complexes.

Sample	Charge Ratio (N:P)	Zeta Potential, mV
pEV.CTL + PG	2.5:1	−5.0 ± 11.5
pEV.Th + PG	2.5:1	−3.3 ± 20.9
pEV.CTL + PAMAM	3:1	−27.3 ± 6.9
pEV.Th + PAMAM	3:1	−9.6 ± 6.7
